# Supplementary figures and images for: Patient-derived monoclonal antibody neutralizes HCV infection in vitro and vivo without generating escape mutants
Source: PLoS One. 2022 Sep 22;17(9):e0274283. doi: 10.1371/journal.pone.0274283 (PMC9499215; doi:10.1371/journal.pone.0274283)

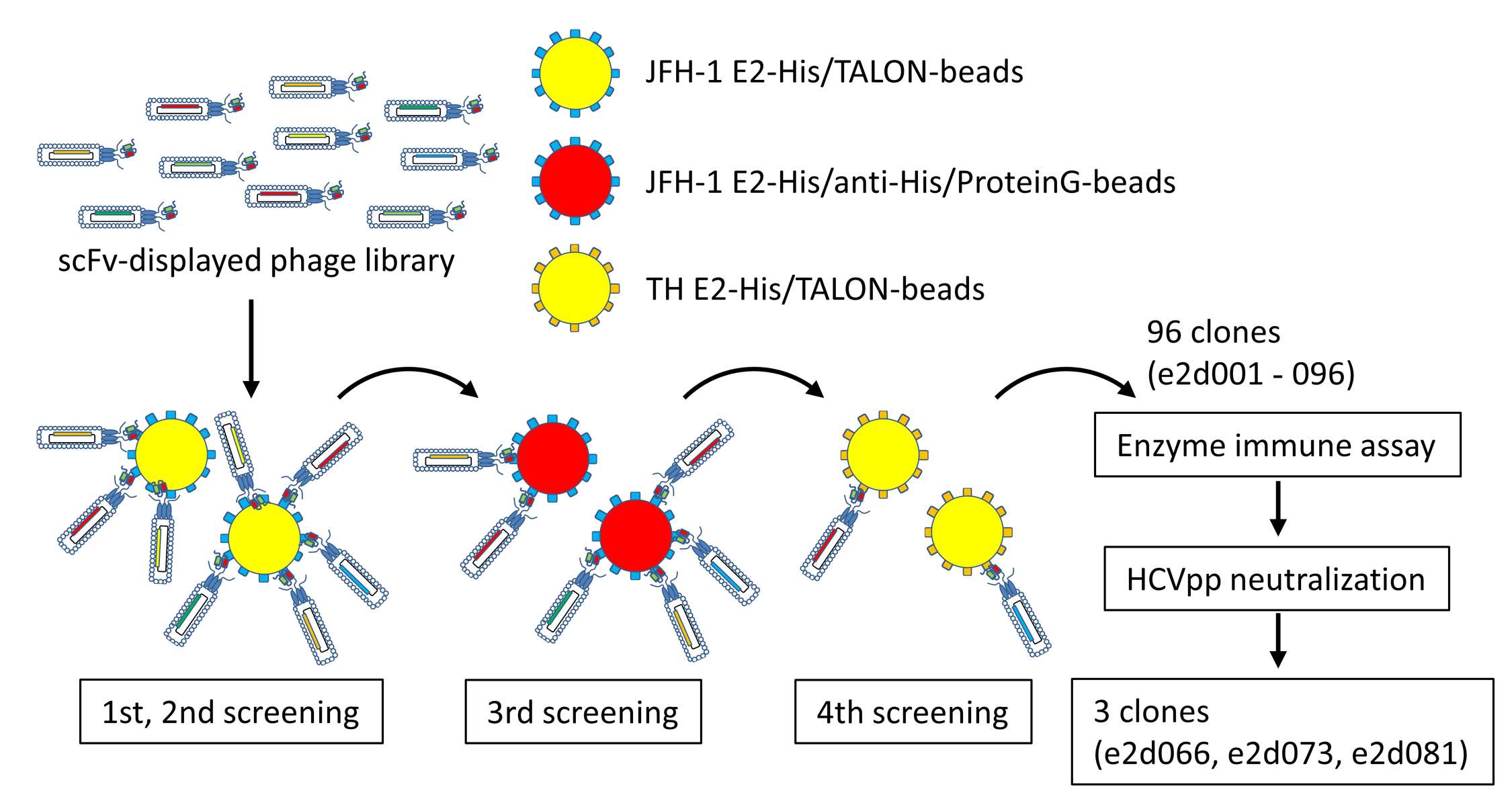

Supplement: S1 Fig — The hepatitis C virus (HCV) E2 protein derived from either strain TH (genotype 1b) or strain JFH-1 (genotype 2a) was allowed to bind to the surface of magnetic beads, and the single chain Fv (scFv) phage library then was added to the coated beads. Specifically bound phages were amplified by infection of E. coli. These steps were repeated for a total of 4 times to enrich for phages with specific binding to the HCV E2 protein. To confirm the specific binding of scFv phages to HCV E2 protein-bound magnetic beads, the gene encoding the corresponding IgG was constructed from phagemid scFv-encoding sequences by PCR cloning. Specific binding of these antibodies to HCV E2 protein was assessed by enzyme immune assay (EIA) recognition and neutralization of HCV pseudoparticle (HCVpp) infection. (TIF) [file pone.0274283.s001.tif]

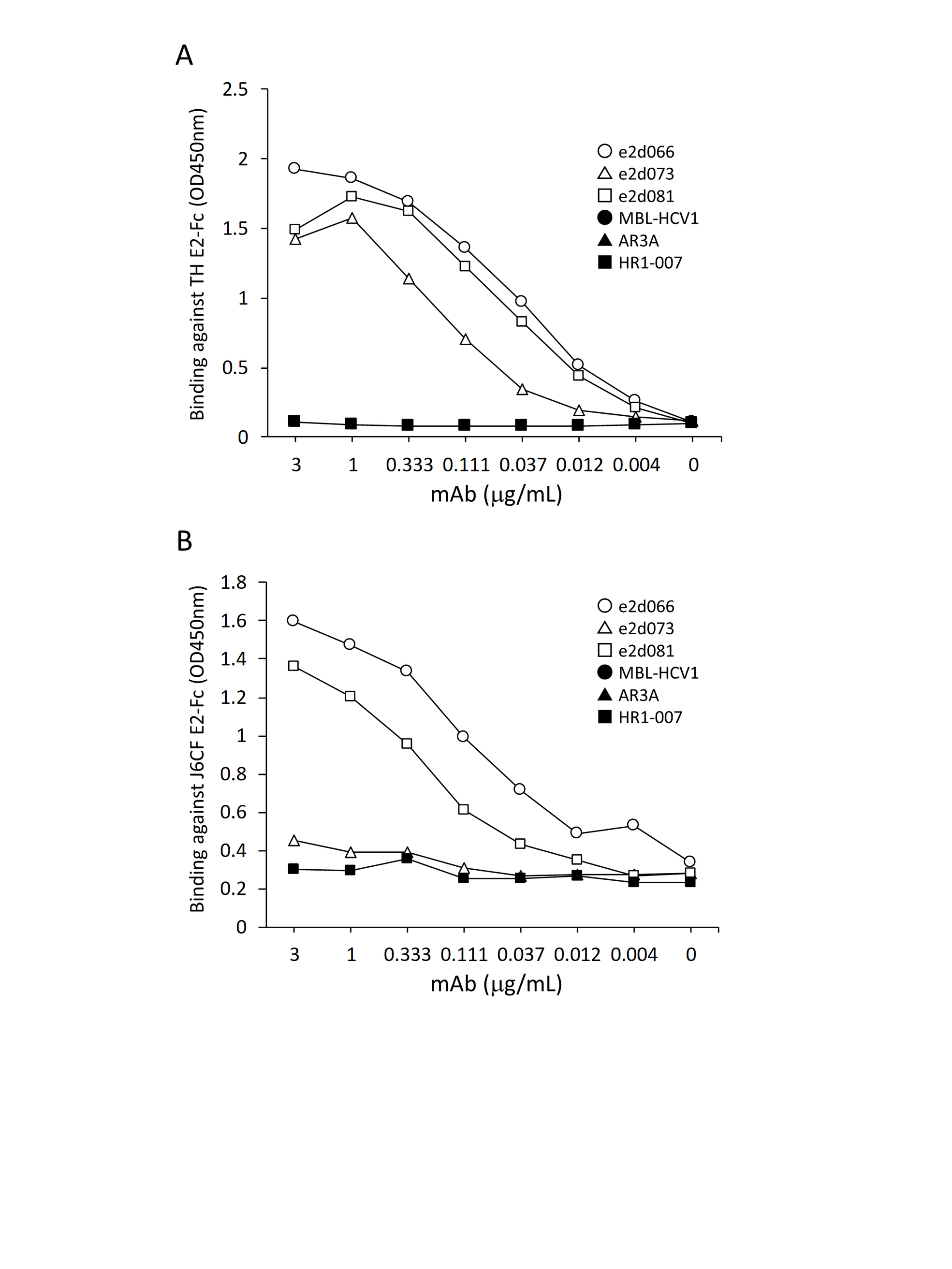

Supplement: S3 Fig — Binding affinity of obtained anti-E2 antibodies to E2 proteins from (A) TH and (B) J6CF was analyzed by enzyme immune assay. The titers of antibodies were measured by a peroxidase assay kit. Open-circle: e2d066 IgG, open-triangle: e2d073 IgG, open-square: e2d081 IgG, filled-square: HR1-007 IgG (control). (TIF) [file pone.0274283.s003.tif]

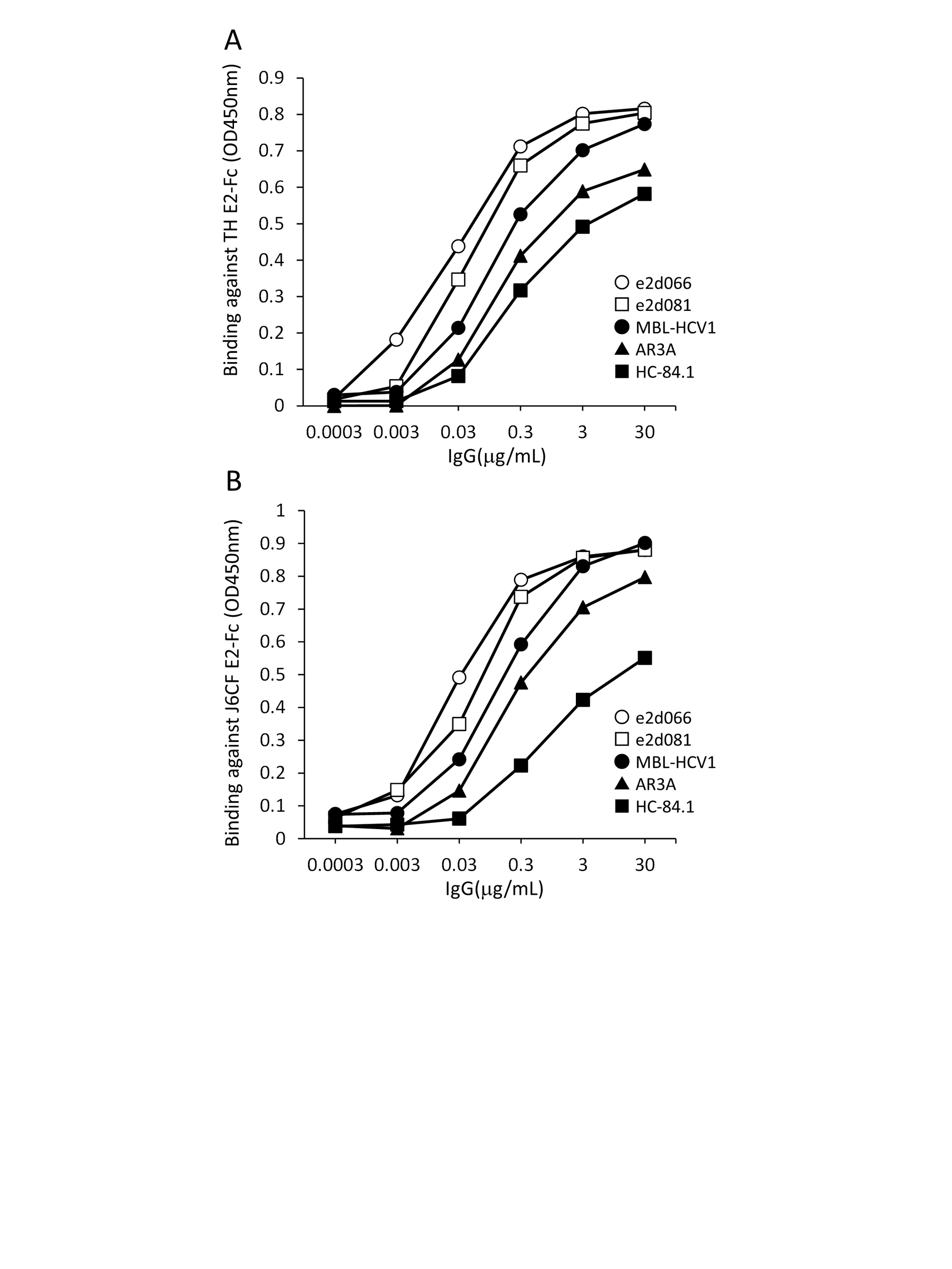

Supplement: S4 Fig — Recombinant E2 proteins (A) TH E2-Fc and (B) J6CF E2-Fc were used to coat Nunc-Immune plates. The prepared plates were used to detect specific bindings of the anti-E2 antibodies (e2d066 IgG, e2d081 IgG, MBL-HCV1, AR3A, and HC-84.1). Horseradish peroxidase-conjugated anti-human immunoglobulin G (IgG-HRP) was used to detect the binding to human IgG. The titers of antibodies were measured using a peroxidase assay kit for ELISA (Sumitomo Bakelite Co., Ltd.). Open-circle: e2d066 IgG, open-square: e2d081 IgG, filled-circle: MBL-HCV1 (linear epitope control antibody), filled-triangle: AR3A (conformational epitope control antibody), filled-square: HC-84.1 (conformational epitope control antibody). (TIF) [file pone.0274283.s004.tif]

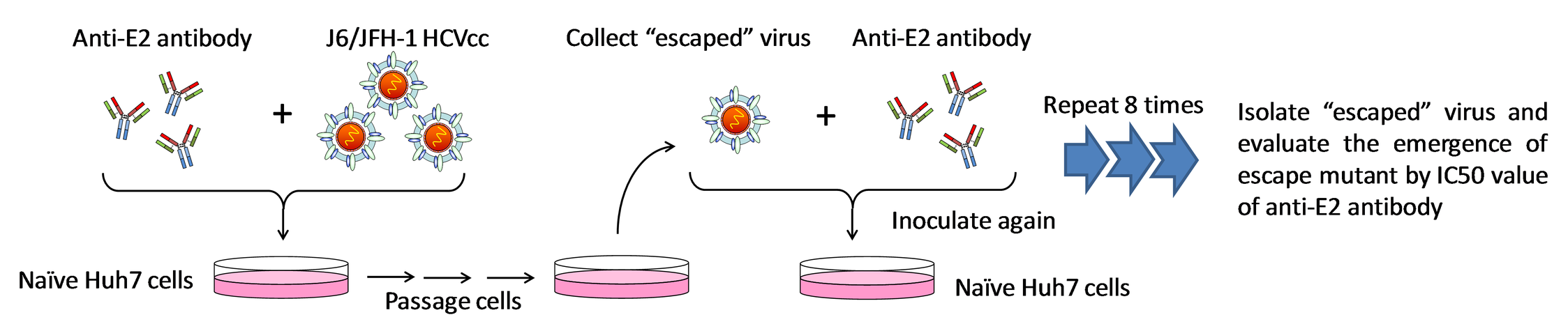

Supplement: S5 Fig — Each IgG antibody was diluted with phosphate-buffered saline (PBS) and combined with J6/JFH-1 cell culture-generated infectious hepatitis C virus particles (HCVcc); the mixture was allowed to react at 37°C for 1 hour. The antibody-HCVcc mixture then was added to Huh7 cells seeded in a 12-well plate to permit infection of the cells with HCVcc. Three days after the infection, the cells were subcultured into a 6-well plate, and the culture supernatant was collected at 3 and 6 days after the start of subculturing. An infectious titer measurement was performed to confirm that the collected culture supernatant contained J6/JFH-1 HCVcc. The culture supernatants that contained J6/JFH-1 HCVcc were used for subsequent infection, such that the collected culture supernatant again was mixed with the antibody, and the mixture was added to uninfected Huh7 cells. These steps were repeated for a total of 8 times. The infection-inhibiting activity of the anti-E2 antibody against the virus isolated at the final round (J6/JFH-1_EM) was determined to assess the possible presence of an escape mutant. (TIF) [file pone.0274283.s005.tif]

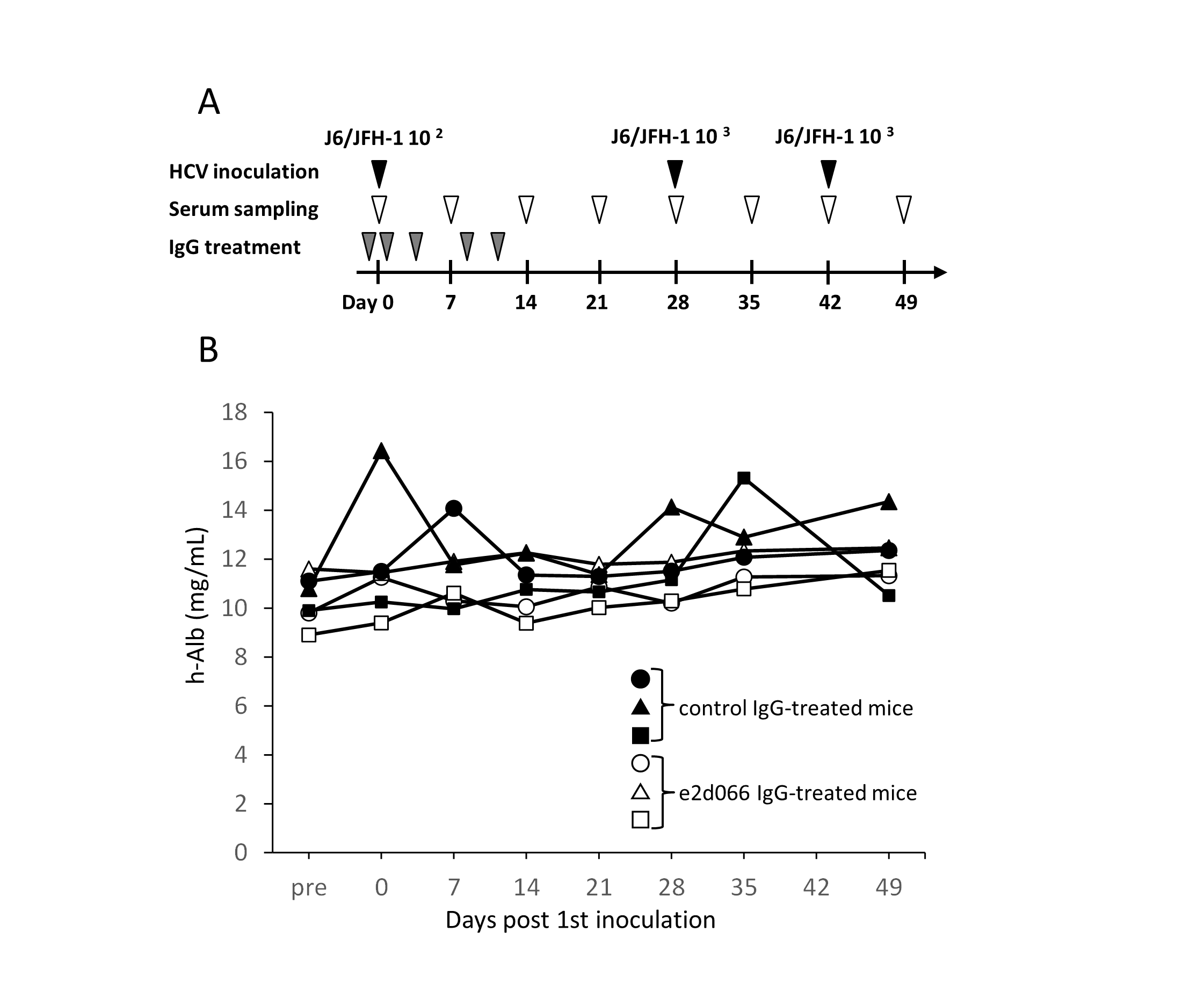

Supplement: S6 Fig — (A) Study design of in vivo infection experiment. Human liver-transplanted immunodeficient mice were inoculated with J6/JFH-1 cell culture-generated infectious hepatitis C virus particles (HCVcc) on Days 0, 28, and 42 (inoculated HCV RNA copy numbers were 102, 103, and 103 per mouse respectively; filled triangles). Anti-E2 antibody e2d066 or control human IgG were administered by intraperitoneal injection (800 μg/mouse, i.p.; gray triangles) to the mice on Day -1 (i.e., one day before) and Days 1, 4, 8, 11 after the first (Day-0) challenge with J6/JFH-1 HCVcc. Blood samples of the mice were collected once weekly (open triangle) and processed to obtain serum sample. (B) Human albumin in the blood of the chimeric mice was measured with the Alb-II Kit (Eiken Chemical, Tokyo, Japan). Filled points: control IgG treated mice. Open points: e2d066 treated mice. (TIF) [file pone.0274283.s006.tif]
