# Supplementary material for: Patient-derived monoclonal antibody neutralizes HCV infection in vitro and vivo without generating escape mutants
Source: PLoS One. 2022 Sep 22;17(9):e0274283. doi: 10.1371/journal.pone.0274283 (PMC9499215; doi:10.1371/journal.pone.0274283)
Supplement: S1 Table — (DOCX) [file pone.0274283.s007.docx]

**S1 Table List of antibodies**

| Antibodies | Supplier | Species | Type | Reference |
| --- | --- | --- | --- | --- |
| e2d066 | In house | human | monoclonal |  |
| e2d073 | In house | human | monoclonal |  |
| e2d081 | In house | human | monoclonal |  |
| E2d066scFv | In house | human | monoclonal |  |
| E2d073scFv | In house | human | monoclonal |  |
| E2d081scFv | In house | human | monoclonal |  |
| MBL-HCV1 | In house | human | monoclonal | Broering et al., J. Viol., 2009, vol. 83, pp. 12473-12482 |
| AR3A | In house | human | monoclonal | Law M., et al., Nat Med, 2008, vol.14, pp 25-27 |
| 8D10-3 | In house | mouse | monoclonal | unpublished |
| HC-84.1 | In house | human | monoclonal | Krey et al., PLOS Pathogens, 2013, vol.9, e1003364 |
| Anti-CD81 (JS-81) | BD Pharmingen | mouse | monoclonal | 555675 |
| 2H9 | In house | mouse | monoclonal | Wakita T. et al., Nat. Med., 2005, vol. 11, pp. 791-796 |
| HRP-labeled anti-human IgG sheep IgG | GE Healthcare | sheep | polyclonal | NA933-1ML |
| HRP-labeled anti-human IgG F(ab)’ goat IgG | Thermo Fisher Scientific | goat | polyclonal | 31483 |
| Alexa Fluoro 488 anti-mouse IgG (H+L) goat IgG | Thermo Fisher Scientific | goat | polyclonal | A11001 |
| HR1-007 | In house | human | monoclonal | Yakushiji et al., Cancer Science, (2019), vol.110, pp 2722-2733 |
