# Supplementary material for: Patient-derived monoclonal antibody neutralizes HCV infection in vitro and vivo without generating escape mutants
Source: PLoS One. 2022 Sep 22;17(9):e0274283. doi: 10.1371/journal.pone.0274283 (PMC9499215; doi:10.1371/journal.pone.0274283)
Supplement: S2 Table — (DOCX) [file pone.0274283.s008.docx]

S2 Table List of primers

【VH amplification】

Human VH primer

GTCCTCGCAACTGCGGCCCAGCCGGCCATGGCC CAGGTGCAGCTGGTGCAGTCTGG

GTCCTCGCAACTGCGGCCCAGCCGGCCATGGCC CAGRTCACCTTGAAGGAGTCTGGTCC

GTCCTCGCAACTGCGGCCCAGCCGGCCATGGCC GAGGTGCAGCTGGTGGAGTCTGG

GTCCTCGCAACTGCGGCCCAGCCGGCCATGGCC CAGGTGCAGCTGCAGGAGTCGGG

GTCCTCGCAACTGCGGCCCAGCCGGCCATGGCC CAGGTGCAGCTACAGCAGTGGGG

GTCCTCGCAACTGCGGCCCAGCCGGCCATGGCC CAGGTACAGCTGCAGCAGTCAGG

GTCCTCGCAACTGCGGCCCAGCCGGCCATGGCC CAGGTGCAGCTGGTGCAATCTGGGTCTGAGT

Human JH primer

AGAACCACCGCGGCCGCTCGAGACGGTGACCAGGGTGC

AGAACCACCGCGGCCGCTCGAGACGGTGACCATTGTCC

AGAACCACCGCGGCCGCTCGAGACGGTGACCAGGGTTC

AGAACCACCGCGGCCGCTCGAGACGGTGACCGTGGTCC

【VLCL amplification】

Human VL primer

GTCCTCGCAACTGCGGCCCAGCCGGCCATGGCC GACATCCAGATGACCCAGTCTCC

GTCCTCGCAACTGCGGCCCAGCCGGCCATGGCC GATGTTGTGATGACTCAGTCTCC

GTCCTCGCAACTGCGGCCCAGCCGGCCATGGCC GAAATTGTGTTGACGCAGTCTCC

GTCCTCGCAACTGCGGCCCAGCCGGCCATGGCC GACATCGTGATGACCCAGTCTCC

GTCCTCGCAACTGCGGCCCAGCCGGCCATGGCC GAAACGACACTCACGCAGTCTCCAGCATT

GTCCTCGCAACTGCGGCCCAGCCGGCCATGGCC GAAATTGTGCTGACTCAGTCTCCAGACTT

GTCCTCGCAACTGCGGCCCAGCCGGCCATGGCC GATGTTGTGATGACACAGTCTCCAGCTTT

GTCCTCGCAACTGCGGCCCAGCCGGCCATGGCC CAGTCTGTGTTGACGCAGCCGCC

GTCCTCGCAACTGCGGCCCAGCCGGCCATGGCC CAGTCTGCCCTGACTCAGCCTGC

GTCCTCGCAACTGCGGCCCAGCCGGCCATGGCC CAGTCTGCCCTGACTCAGCCTC

GTCCTCGCAACTGCGGCCCAGCCGGCCATGGCC TCCTATGAGCTGACTCAGCCAC

GTCCTCGCAACTGCGGCCCAGCCGGCCATGGCC TCTTCTGAGCTGACTCAGGACCC

GTCCTCGCAACTGCGGCCCAGCCGGCCATGGCC CAGCCTGTGCTGACTCAATCATC

GTCCTCGCAACTGCGGCCCAGCCGGCCATGGCC CAGCTTGTGCTGACTCAATCGCC

GTCCTCGCAACTGCGGCCCAGCCGGCCATGGCC CTGCCTGTGCTGACTCAGCCCCC

GTCCTCGCAACTGCGGCCCAGCCGGCCATGGCC CAGGCTGTGCTCACTCAGCCGTC

GTCCTCGCAACTGCGGCCCAGCCGGCCATGGCC CAGCCTGTGCTGACTCAGCCAHCTTCC

GTCCTCGCAACTGCGGCCCAGCCGGCCATGGCC CAGGCTGTGCTGACTCAGCCGGCTTCC

GTCCTCGCAACTGCGGCCCAGCCGGCCATGGCC AATTTTATGCTGACTCAGCCCCA

GTCCTCGCAACTGCGGCCCAGCCGGCCATGGCC CAGRCTGTGGTGACTCAGGAGCCCTCACTG

GTCCTCGCAACTGCGGCCCAGCCGGCCATGGCC CAGACTGTGGTGACCCAGGAGCCATCGTTC

GTCCTCGCAACTGCGGCCCAGCCGGCCATGGCC CAGCCTGTGCTGACTCAGCCACCTTCTGCA

GTCCTCGCAACTGCGGCCCAGCCGGCCATGGCC CAGGCAGGGCTGACTCAGCCACCCTCGGTG

hCLASC

TCGACTGGCGCGCCGAACACTCTCCCCTGTTGAAGCTCTTTGTG

TCGACTGGCGCGCCGAACATTCTGTAGGGGCCACTGTCTTCTC
