# Supplementary material for: Patient-derived monoclonal antibody neutralizes HCV infection in vitro and vivo without generating escape mutants
Source: PLoS One. 2022 Sep 22;17(9):e0274283. doi: 10.1371/journal.pone.0274283 (PMC9499215; doi:10.1371/journal.pone.0274283)
Supplement: S3 Table — (DOCX) [file pone.0274283.s009.docx]

**S2 Table IC50 values of antibodies against HCVpp infection**

| IC50 value (μg/mL) | | |
| --- | --- | --- |
| Clone No. | TH HCVpp | J6 HCVpp |
| e2d066 | 1.55 | 12.49 |
| e2d073 | 4.39 | 48.52 |
| e2d081 | 1.06 | 23.54 |

IC50; 50 % infection inhibiting concentration

HCVpp; HCV pseudoparticles
