# Supplementary material for: Patient-derived monoclonal antibody neutralizes HCV infection in vitro and vivo without generating escape mutants
Source: PLoS One. 2022 Sep 22;17(9):e0274283. doi: 10.1371/journal.pone.0274283 (PMC9499215; doi:10.1371/journal.pone.0274283)
Supplement: S4 Table — (DOCX) [file pone.0274283.s010.docx]

**S3 Table List of CDRs displayed by the scFv phages**

| VH chain |  |  |  |
| --- | --- | --- | --- |
| Clone No. | CDR1 | CDR2 | CDR3 |
| e2d066, e2d081 | SYAVN | RIMPLVGLPEYAERFQE | GVMKIFGEVPLNLDF |
| e2d073 | SFAID | RIIPIADVSDYAQKFQG | SPMLTFGGPNAFGAFDV |
|  |  |  |  |
| VL chain |  |  |  |
| Clone No. | CDR1 | CDR2 | CDR3 |
| e2d066 | TGTSDNVGSYRTVS | DVNKRPS | SSYTRSSSLA |
| e2d073 | SGTSSNIGDNYVS | DNNKRPS | GTWDSSLSSVV |
| e2d081 | RASQSISSYLN | AASSLQS | QQSYSTPQFT |

CDR; complementarity determining region

scFv; single-chain variable fragment

VH; variable domain, heavy chain

VL; variable domain, light chain
