# Supplementary material for: Patient-derived monoclonal antibody neutralizes HCV infection in vitro and vivo without generating escape mutants
Source: PLoS One. 2022 Sep 22;17(9):e0274283. doi: 10.1371/journal.pone.0274283 (PMC9499215; doi:10.1371/journal.pone.0274283)
Supplement: S5 Table — (DOCX) [file pone.0274283.s011.docx]

**S4 Table IC50 value of HC-84.1 antibody against escape mutant HCVcc**

| Escape mutant viruses | IC50 (μg/ml) for the HC-84.1 antibody* |
| --- | --- |
| J6/JFH-1 EM_control** | <0.01 |
| J6/JFH-1 EM_HC-84.1 | 4.663 |

IC50; 50 % infection inhibiting concentration.

HCVcc; Cell culture-generated infectious HCV particles

* IC50 (μg/ml) for HC-84.1 antibody against the indicated escape viruses was determined by focus forming assay.

** Unrelated control antibody was used to raise this virus.
